# Preparation of UV Debonding Acrylate Adhesives by a Postgrafting Reaction

**DOI:** 10.3390/ma16175911

**Published:** 2023-08-29

**Authors:** Juan Wang, Zhikai Dong, Jingwen Chen, Shuangjun Chen

**Affiliations:** 1 College of Materials Science and Engineering, Nanjing Tech University, Nanjing 210009, China; 2 Suqian Advanced Materials Institute, Nanjing Tech University, Suqian 223800, China

**Keywords:** UV debonding adhesives, acrylates, postgrafting, cross-linking, rheology

## Abstract

UV debonding acrylate adhesive (UDAA) plays a crucial role in the semiconductor industry, where its excellent adhesion is required to ensure the stability of silicon wafers and leave no residue on the surface after UV irradiation. The necessary UV debonding is achieved through the formation of rigid networks by the reactions of all the vinyl groups in the system. Acrylate copolymers with vinyl groups are typically obtained by the grafting reaction of isocyanate with a side-chain hydroxyl comonomer. However, these grafting reactions easily fail due to early cross-link formation. In this study, we illustrate a straightforward method for preparing UDAA by conducting a postgrafting reaction after one-step mixing of isocyanate functional monomer (IPDI-H) and hydroxyl acrylate copolymers (BA-H), thereby skipping the abovementioned vinyl grafting process. The chemical structures of the synthesized IPDI-H and BA-H were confirmed using Fourier transform infrared spectroscopy (FTIR) and proton nuclear magnetic resonance (^1^H-NMR) analysis. Gel permeation chromatography (GPC) was employed to determine their molecular weights, while differential scanning calorimetry (DSC) was used to determine their glass transition temperatures. The postgrafting reactions successfully introduced vinyl groups onto the polyacrylate copolymer chains, resulting in high bonding strength during use and a significant decrease in peeling strength after UV irradiation. Rheological methods, including the three-interval thixotropy test (3ITT) and tack test modes, were employed to characterize a series of acrylate UV debonding adhesives. The recovery percentage of the storage modulus in the 3ITT mode indicated that a 0.6 wt% isocyanate curing agent made the UV debonding adhesives resistant to deformation. From the maximum normal force in the tack test mode, it was found that UDAA with 10 wt% PETA monomer and 30 wt% C5 tackifying resin exhibited excellent combined adhesion and debonding properties, which were further confirmed by peel strength tests. Microscope images of the wafer surfaces after removing the adhesive tapes demonstrated the excellent UV debonding properties achieved after 40 s of UV irradiation through the postgrafting reaction. The prepared UDAA has excellent properties; the peel strength can reach 15 N/25 mm before UV irradiation and can be reduced to 0.5 N/25 mm after ultraviolet irradiation. This research establishes a comprehensive method for understanding and applying UDAA in various applications.

## 1. Introduction

Ultraviolet (UV) debonding adhesive (UDA) is a vital adhesive [1,2] in the semiconductor industry, particularly during the cutting stage after silicon wafer grinding and chip processing [3,4]. UDAs are required to have a strong adhesion force to ensure wafer stability during cutting or polishing. Simultaneously, after UV irradiation, the adhesive must be peeled off the wafer surface without leaving any residue to facilitate subsequent processes. Balancing these two requirements presents a significant challenge in developing high-quality UDA materials.

Compared to other adhesive types [5], acrylate adhesives offer several advantages. These include high transparency, exceptional optical clarity resulting from polymer compatibility and resistance to yellowing, a well-balanced combination of adhesion and cohesion, and excellent water resistance [6,7,8,9,10]. Seung-Rak Son et al. employed oxime-based photoinitiators to create water-resistant and highly adhesive acrylate-based sealing materials for hermetic optical devices. The results demonstrated that acrylate-based sealants exhibit the strongest adhesion and the lowest water permeability [11]. Xiaoyong Zhang et al. utilized acrylate monomers and maleimide (Mal) to synthesize pressure-sensitive adhesives (PSAs), achieving significantly enhanced adhesion performance and thermal stability of acrylate copolymers [12].

UV Debonding Acrylate Adhesive (UDAA) exhibits high adhesion when utilized; however, its adhesive strength rapidly decreases upon UV irradiation. UADD is widely used in the acid treatment protection of screen glass such as mobile phones and pads, the grinding and cutting of silicon wafers, the cutting process fixation of various packaging parts, and the cutting process fixation of optical glass. In their study, Han et al. synthesized a copolymer with a hydroxyl group by employing 2-ethylhexylacrylate, methyl methacrylate, acrylate acid, and hydroxyethyl acrylate monomer through free radical polymerization. They then mixed the copolymers with a photoinitiator and aziridine crosslinker to prepare a photocrosslinkable adhesive. The initial adhesion strength was notably high due to the carboxyl and urethane groups, which possess a strong affinity for substrates. However, after UV irradiation, the peel strength declined sharply as a result of the cross-linking of methacrylate groups [13]. Minkyu Kim et al. employed an oligomer, namely poly(sodium methacrylate sulfonate) (pSBMA), along with catechol functional groups and photocleavable crosslinkers, within a single polymer structure to synthesize photosensitive trimeric adhesives. These adhesives demonstrated significant bond strength when adhered to polyester films. However, the bond strength markedly decreased after a brief exposure to UV radiation due to the rapid photodegradation of the nitrobenzyl ester-containing crosslinker [14].

Moreover, the residual amount of adhesives is closely related to the bond strength, and the adhesive properties can be evaluated through tack test modes in rheological methods. Shiho Kawashima et al. utilized the tack test to assess the influence of purified attapulgite clays on the adhesive properties of cement pastes. Their results indicated that the tack test is a suitable method for obtaining valuable information regarding the adhesive properties and structural evolution of the material [15].

In this study, a series of UDAAs were synthesized with the capability of debonding after UV irradiation [16]. The chemical structures of these UDAAs were elucidated using Fourier transform infrared spectroscopy (FTIR) and nuclear magnetic resonance of protons (^1^H-NMR). Gel permeation chromatography (GPC) and differential scanning calorimetry (DSC) were employed to determine the molecular weight and glass transition temperatures of BA-H, respectively. Additionally, rheological methods, including the interval thixotropy test (3ITT) and tack test modes, were utilized to characterize their bonding and debonding properties [17,18]. Microscope images of the wafer surfaces, obtained after peeling off the tapes, were analyzed to provide evidence of the debonding effects. The experimental results indicate that the prepared UDAA has excellent properties; the peel strength can reach 15 N/25 mm before UV irradiation and can be reduced to 0.5 N/25 mm after ultraviolet irradiation. Compared with the properties of UV debonding adhesives used in industry (before UV irradiation: 12~20 N/25 mm; after UV irradiation: 0.2~0.5 N/25 mm) and UV debonding adhesives recorded in literature (before UV irradiation: 13.3 N/25 mm; after UV irradiation: 0.2 N/25 mm) [13], the properties of UDAA prepared in this study have met the requirements of market applications; however, the properties can still be further optimized.

## 2. Experimental

### 2.1. Materials

Isophorone diisocyanate (IPDI) and pentaerythritol triacrylate (PETA) were procured from Shanghai Macklin Biochemical Technology Co., Ltd. (Shanghai, China). Butyl acrylate (BA), hydroxyethyl methacrylate (HEMA), hydroxypropyl methacrylate (HPMA), and methyl methacrylate (MMA) were sourced from Shandong Baihong New Material Co., Ltd. (Shandong, China). The catalyst dibutyltin dilaurate (DBTDL, 95%) and photoinitator 2-hydroxy-2-methylpropiophenone (HMPP) were respectively purchased from Shanghai Aladdin Biochemical Technology Co., Ltd. (Shanghai, China). Azobisisobutyronitrile (AIBN) was obtained from Sigma-Aldrich (Buchs, Switzerland), and the solvent ethyl acetate was purchased from Wuxi Yasheng Chemical Company (Wuxi, China). Hydroquinone, used as an inhibitor, was obtained from Sinopharm Chemical Reagent Co., Ltd. (Beijing, China). The isocyanate curing agent (Desmodur N75, polyisocyanate oligomer) was supplied by Changzhou Institute of Coatings and Chemicals (Changzhou, China). Tackifying resin was purchased from Shenzhen Jitian Chemical Co., Ltd. (Shenzhen, China). All chemicals were used as received.

### 2.2. Synthesis and Preparation

#### 2.2.1. Samples

A total of 22.23 g of IPDI (0.10 mol, monomer, introduction of isocyanate groups), 0.19 g of DBTDL (0.5 wt% of IPDI and HEMA reactants, catalyst), 0.04 g (0.1 wt%, inhibitor) of hydroquinone, and 26.10 g (40 wt%) of solvent ethyl acetate were added to a three-necked flask equipped with a reflux condenser. The reaction temperature was raised to 50 °C under a nitrogen atmosphere, after which 16.92 g (0.13 mol) of HEMA (a monomer with a molar ratio of HEMA to IPDI of 1.30:1) was added dropwise with magnetic stirring and maintained for 7 h in a thermoregulated oil bath. The product was sealed and stored in a drying dish. The synthesis mechanism of this step was the reaction between the isocyanate group (-NCO) in the straight chain of IPDI and the hydroxyl group (-OH) in HEMA. The obtained samples were designated as isocyanate functional monomers (IPDI-H) because they contained isocyanate groups.

The preparation of the acrylate copolymer was performed by free radical polymerization in a three-necked flask equipped with a mechanical agitator. Firstly, 111.46 g of BA (0.87 mol, soft monomer), 5.01 g of MMA (0.05 mol, hard monomer), 2.60 g of HEMA (0.02 mol, hydroxyl monomer), 8.65 g of HPMA (0.06 mol, hydroxyl monomer), 1.28 g (0.10 wt% relative to the above monomers) of AIBN, and 237.20 g (65.0 wt% relative to the above monomers, solvent) of ethyl acetate were added to the flask. The molar ratio of BA/MMA/HEMA/HPMA remained 87:5:2:6. Furthermore, the mixture was heated to 80 °C under a continuous nitrogen atmosphere, and the reaction was continuously stirred for 8 h. Finally, the acrylate copolymer was transferred into a prepared brown glass bottle and allowed to cool to room temperature. The obtained samples were designated as acrylate copolymers (BA-H). The solid content of BA-H was determined to be 35.91 wt%. By extending the polymerization time and increasing the temperature during the final stages of the reaction, the residual ratio of unreacted monomers was reduced.

#### 2.2.2. Preparation

After cooling the acrylate copolymer, which had a solid content of 35.91 wt%, to room temperature, the following components were added to the beaker: 20.00 g of BA-H, 2.08 g of isocyanate functional monomer (IPDI-H), 0.07 g of DBTDL (1.0 wt% relative to BA-H), 0.72 g of pentaerythritol triacrylate (PETA, 10.0 wt% relative to BA-H), 0.04 g of isocyanate curing agent (Desmodur N75, isocyanate curing agent, 0.6 wt% relative to BA-H), 0.29 g of 2-hydroxy-2-methylpropiophenone (HMPP, photoinitiator, 4.0 wt% relative to BA-H), and 1.19 g of tackifying resin (30 wt% C5 tackifying resin). The mixtures were stirred continuously to ensure thorough and even mixing, resulting in the preparation of UDAAs. However, the specific formulations for UDAAs can be found in Section 3.

**Figure 1:**
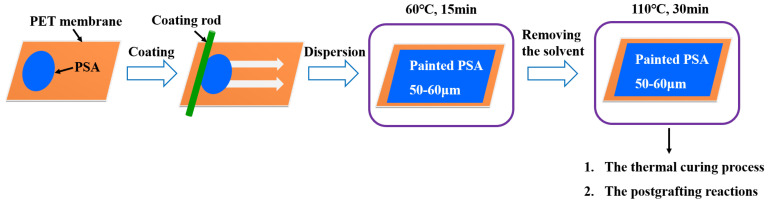
The procedure of IPDI-H and BA-H postgrafting reactions and the thermal curing process.

The preparation of UDAAs involved the following experimental methods. The process of IPDI-H and BA-H postgrafting reactions, in which the vinyl groups of IPDI-H were grafted onto the main chains of BA-H, as well as the thermal curing process, is illustrated in Figure 1. In summary, acrylate UV debonding adhesives with a thickness of 50–60 μm (50–60 µm after solvent evaporation, based on the solid content) were applied to PET films using a coating machine. The samples underwent two stages of processing. First, the coated PET films were thermally cured in a blast oven at 60 °C for 15 min to evaporate the solvent. Subsequently, the PET films were subjected to postgrafting reactions and a thermal curing process in a vacuum oven at 110 °C for 30 min.

### 2.3. Fourier Transform Infrared Spectroscopy (FTIR)

IR spectra were acquired in IPDI-H and BA-H on a Nicolet IS5 FTIR (Thermo Fisher Scientific, Waltham, MA, USA). The characterization was conducted in the wavenumber range from 4000 to 400 cm^−1^, with a resolution of 4 cm^−1^ in transmission mode. Additionally, all IR spectra were modified by means of baseline correction.

### 2.4. Nuclear Magnetic Resonance of Protons (^1^H-NMR)

^1^H-NMR spectra of the synthesized IPDI-H and BA-H were obtained by using a Bruker DRX-400 (400 MHz for ^1^H and 100 MHz for ^13^C) spectrometer (Rheinstetten, Germany). Approximately 10 mg of the synthetic products IPDI-H and BA-H were dissolved in dimethyl sulfoxide-d6 and deuterated chloroform, respectively, for ^1^H-NMR characterization.

### 2.5. Gel Permeation Chromatography (GPC)

The molecular weights and polydispersity indices of the synthesized polymers were determined by gel permeation chromatography (GPC) in tetrahydrofuran (THF) solvent using PL-GPC 120 (Polymer Laboratories, Shropshire, UK) (25 °C, flow rate 1 mL/min). Samples were dissolved in THF (0.15% g/mL) and passed through a 0.2 μL filter before injection.

### 2.6. Differential Scanning Calorimetry (DSC) and Thermogravimetry Analysis (TGA)

The glass transition temperatures (Tg) of the synthetic products were measured on a DSC (Q20TA Instrument, Newcastle, WA, USA) under a nitrogen atmosphere. For each measurement, the weight of the sample was approximately 6 mg. The sample was cooled to −70 °C at a heating rate of 5 °C/min and maintained for another 1 min to eliminate the heat history. Then, the sample was heated to 60 °C with a heating rate of 5 °C/min. The glass transition temperature was recorded in the heating scan. The thermal stability of the products was tested by thermogravimetry. For each measurement, the weight of the sample was approximately 10 mg. The sample was heated to 810 °C with a heating rate of 10 °C/min.

### 2.7. Rheological Behaviour of Adhesives

Rheological characterization was conducted at room temperature on an Anton Paar MCR 302 rheometer (Anton Paar Instruments, Graz, Austria). The rheometer was equipped with a disposable fixture (d = 25 mm) separated by 1.0 mm.

The 3ITT mode was used for testing the thixotropic recovery performance of UV debonding acrylate adhesives and was divided into three stages. The first stage of 3ITT was 60 s, and this stage was the rest phase: a constant, small shear load is applied to the acrylate UV debonding adhesive. Then, the second stage was the shear phase, where there was a constant larger shear load, and the third stage was the structural recovery process. The thixotropic recovery rate was the recovery ratio of the structural strength at a specified time point in the structural recovery stage. In addition, the thixotropic recovery rate of the sample at 30 s after deformation can be determined by the following equation [17].

% Recovery (%Rec) = G30/G_i_ × 100 (1)

where G_i_ represents the initial G_0_ value of the sample and G_30_ represents the G_0_ values of the samples within the first 30 s after deformation.

A time test was used to monitor the postgrafting reaction. Tack test mode was an adhesion test, which can measure the adhesion value (Fn) of UV debonding acrylate adhesives. The 3ITT test conditions are given as follows: for interval 1, the test was performed at amplitude γ = 0.1% and frequency f = 10 Hz; for interval 2, deformation was performed at amplitude γ = 500% and frequency f = 10 Hz; and the condition of interval 3 (structural regeneration) was the same as that of interval 1. Time test mode tests at amplitude γ = 1%, frequency f = 1 Hz, and temperature T = 70 °C. Tack test mode tests were conducted at normal force F_N_ = 1 N and speed v = 5 mm/s.

### 2.8. Peel Strength

The adhesion of acrylate UV debonding adhesives was evaluated through peel strength tests in accordance with GB/T2792-2014 standards [19]. The tests were conducted using an Instron 4411 universal testing machine (Instron Ltd., Buckinghamshire, UK) at a pulling rate of 300 mm/min. Prior to testing, a coating machine was employed to apply 50–60 µm thick adhesives (based on the solid content, post-solvent evaporation) onto flexible PET film substrates. Subsequently, the PET film was subjected to a preheating process at 60 °C for 15 min in an oven. Following the removal of solvents, the adhesive-coated PET film was heated to 110 °C for 30 min to initiate the postgrafting reaction and heat curing. Next, a release film was applied to the PET film, which was subsequently cut into 15 cm × 2.5 cm splines. These splines were then laminated onto stainless steel (SUS 304) plates and pressed onto a 20 cm × 20 cm, 2 kg iron plate for 30 min. Each condition was tested five times, and the average values were calculated.

### 2.9. Light Microscopy

The residues of copolymer acrylate UV debonding adhesives on the surface of a silicon wafer after peeling were examined using a polarizing microscope system (NP-800TRF). The preparation of samples for residue testing after peeling was as follows: the acrylate UV debonding adhesive was uniformly applied onto a PET film using a 10 μm glue stick coater, and then the film was placed in a 55 °C oven for 10 min. An approximately 2.50 cm^2^ silicon wafer was pressed onto the adhesive-coated PET film with a weight of 500 g for 2 min, followed by curing in a 100 °C oven for 10 min.

## 3. Results and Discussion

### 3.1. Preparation of IPDI-H and BA-H

**Figure 2:**
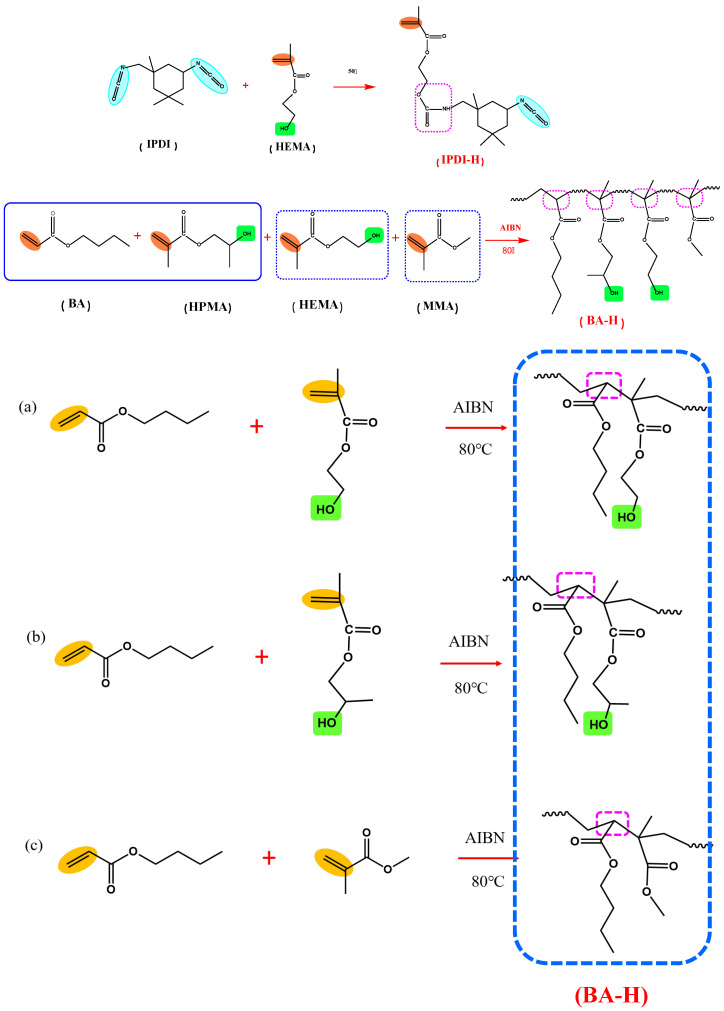
The synthesis routes of the products: IPDI-H(isocyanate functional monomer), BA-H(acrylate copolymers).

**Figure 3:**
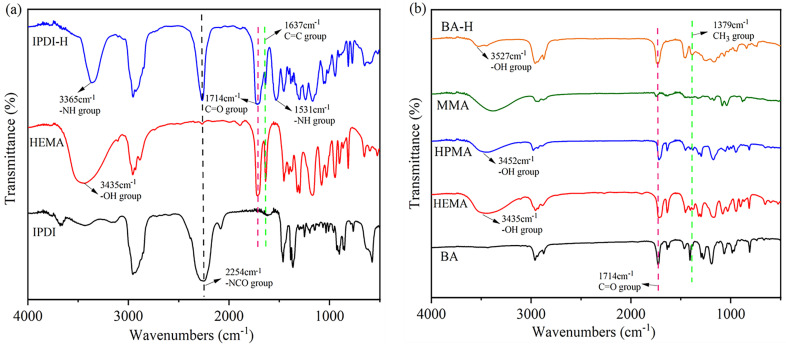
The FTIR spectra for: (**a**) pure IPDI, pure HEMA, and synthetic product IPDI-H; (**b**) pure BA, pure HEMA, pure HPMA, pure MMA, and synthetic product BA-H.

**Figure 4:**
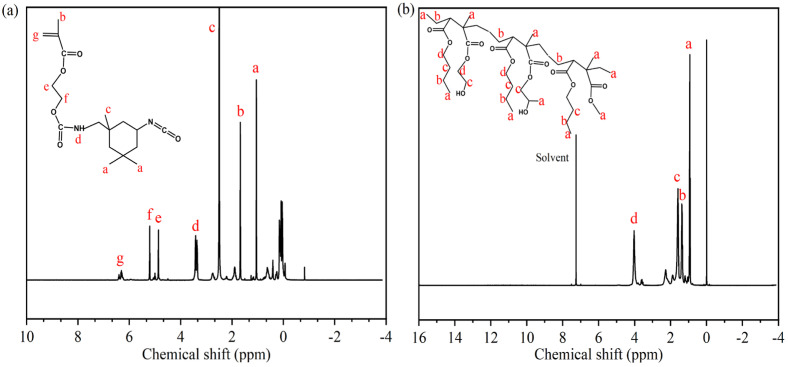
^1^H-NMR spectra of synthetic products: (**a**) IPDI-H, (**b**) BA-H.

**Figure 5:**
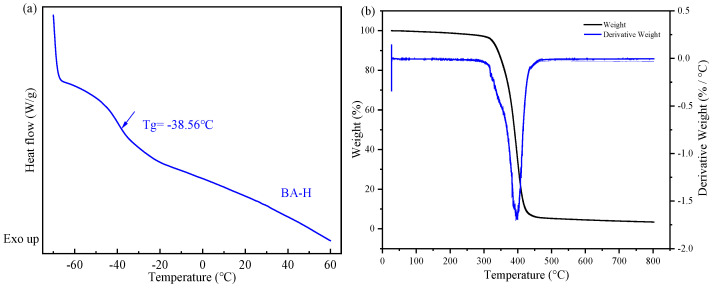
(**a**) the differential scanning calorimetry (DSC) curve of BA-H, the inflection point is marked with an arrow and is attributed to the glass transition; (**b**) the thermogravimetry analysis (TGA) curve of BA-H.

The synthesis pathways for IPDI-H and BA-H are displayed schematically in Figure 2. FTIR and ^1^H-NMR were employed to verify the chemical composition of the synthesized IPDI-H and BA-H, respectively. GPC was utilized to determine the molecular weight of BA-H, while DSC was employed to ascertain the glass transition temperature of BA-H. The outcomes are presented in Figure 3, Figure 4 and Figure 5.

Figure 3a depicts the IR spectra of pure IPDI, pure HEMA, and the synthesized product IPDI-H, which was obtained by the reaction between IPDI and HEMA. An absorption peak at 2254 cm^−1^ was observed for the -N=C=O group [20] in IPDI. Additionally, a peak at 3435 cm^−1^, associated with the -OH stretching of HEMA, was observed. The -NCO group of the straight chain of IPDI preferentially reacts with the hydroxyl group on HEMA due to the higher reactivity of the substituents on the straight chain compared to those on the alicyclic chain [21]. The presence of peaks at 1531 cm^−1^ (-NH group) and 1714 cm^−1^ (C=O group) in IPDI-H [22] indicates successful bonding between HEMA and IPDI, resulting in the formation of urethane bonds (-NHCOO-).

Furthermore, in Figure 3b, the IR spectra of pure BA, pure MMA, pure HEMA, pure HPMA, and the synthesized product BA-H (formed by BA, MMA, HEMA, and HPMA) are presented. A characteristic peak at 3527 cm^−1^ in the BA-H IR spectra can be attributed to the -OH groups in HEMA and HPMA [23,24,25,26]. Additionally, the spectra of BA, MMA, HPMA, and HEMA exhibit a peak at 1637 cm^−1^, corresponding to the -C=C bonds. The disappearance of the -C=C peak in the BA-H product indicates the successful polymerization of BA, MMA, HPMA, and HEMA. Therefore, the aforementioned monomers were effectively polymerized.

The chemical structures of synthesized IPDI-H and BA-H were analyzed using ^1^H-NMR spectroscopy. In Figure 4a, characteristic signals were observed at δa = 1.88 ppm and δc = 3.30 ppm [27], indicating the presence of methyl protons connected to the alicyclic and carbon atoms, respectively. The signals at δe = 5.71 ppm and δf = 6.05 ppm [28] were attributed to the methylene protons, while the hydrogen protons on the double bond appeared at δg = 7.13 ppm [29]. The signal at δd = 4.22 ppm corresponded to the carbamate protons (-NHCOO), supporting the reaction between the isocyanate and the hydroxyl group [30,31]. In Figure 4b, the ^1^H-NMR spectrum of BA-H exhibited a clear peak at approximately δd = 4.03 ppm, which corresponded to the chemical shift of the methylene protons adjacent to the -COO group. Other methylene groups showed chemical shifts around δb = 1.38 ppm and δc = 1.59 ppm. Furthermore, signals at approximately δa = 0.94 ppm were associated with the methyl proton [32].

The GPC results for BA-H are summarized below. The number average molecular weight (Mn) of BA-H is 1.68 × 10^4^ g/mol, and the weight average molecular weight (Mw) is 4.42 × 10^5^ g/mol. However, the Mw exceeds 4.0 × 10^5^ g/mol, indicating a relatively high average molecular weight. At the same time, the polydispersity index (D) was determined to be 26.27, indicating a wide molecular weight distribution. In adhesive applications, low molecular weights are not desirable as they result in low peel strength, fast sticking force, and cohesive damage upon peeling. The adhesive’s performance improves with increasing molecular weight; however, excessively high molecular weights may hinder wetting and dispersion. Therefore, it is necessary to control the molecular weight of the acrylate adhesive within a certain range. Additionally, copolymers with a wide distribution exhibit enhanced adhesion properties [33]. Based on the GPC results, the synthesized BA-H meets the requirements of a specific molecular weight and wide distribution.

In Figure 5a, the DSC method was employed to detect the glass transition temperature (Tg) of the acrylate copolymer (BA-H). The Tg of BA-H was determined to be −38.56 °C, which closely aligns with the typical value for polybutyl acrylate. The soft monomer butyl acrylate (BA) has a Tg of approximately −56 °C and a molecular weight of 128.119 Da. Additionally, the hard monomer methyl methacrylate (MMA) exhibits a Tg of 105 °C and a molecular weight of 100.12 Da [34]. Furthermore, the Tg and molecular weight values of the hydroxyacrylate hard monomers hydroxyethyl methacrylate (HEMA) and hydroxypropyl methacrylate (HPMA) were found to be 55 °C, 70 °C, 130.14 Da, and 144.17 Da [35], respectively. Considering that the molar ratio of BA, MMA, HEMA, and HPMA in the synthesis of BA-H was 87:5:2:6, the polymer’s Tg was calculated as −39.05 °C using the FOX formula, which closely matched the experimental results. In Figure 5b, all the weight loss was concentrated in a relatively narrow range above 300 °C. BA-H did not show significant weight loss until above 300 °C, indicating it has excellent thermal stability.

The glass transition temperature of the polymer (Tg, °C) was calculated by the Equation (2):

(2) $$\frac{1}{Tg}=\frac{\omega 1}{Tg1}+\frac{\omega 2}{Tg2}+\ldots +\frac{\omega n}{Tgn}$$

*Tg*—the glass transition temperature of the copolymer, °C;
*ω_n_*—the mass fraction of each monomer involved in copolymerization;
*Tgn*—the glass transition temperature of each monomer used in copolymerization, °C.

### 3.2. The Postgrafting Reaction of IPDI-H and BA-H

**Figure 6:**
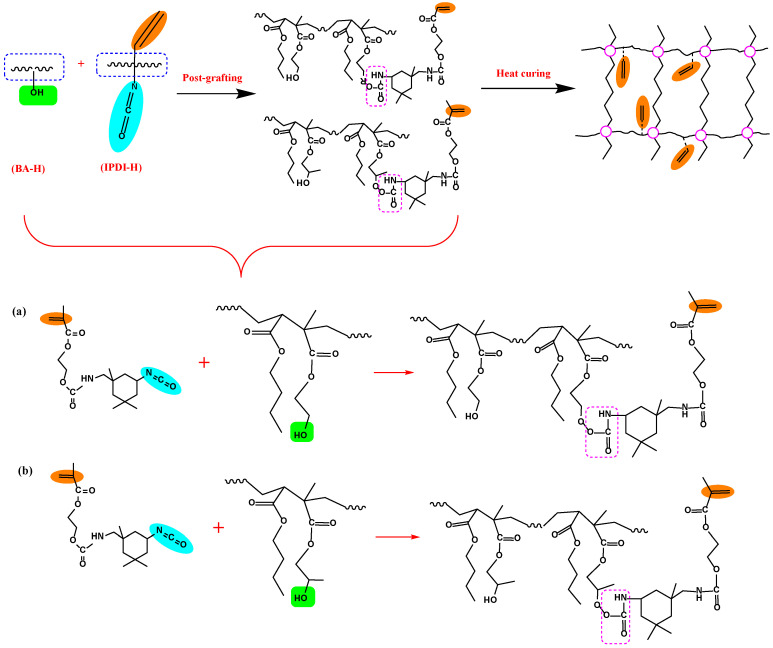
Schematic mechanism of the postgrafting reaction between IPDI-H and BA-H and the heat-curing crosslinking of UDAAs, (**a**) the reaction of IPDI-H with HEMA; (**b**) the reaction of IPDI-H with HEMA.

The synthesis routes for IPDI-H and BA-H postgrafting reactions and thermal curing are illustrated in Figure 6. The objective of the “postgrafting” step is to facilitate a reaction between the -NCO groups in IPDI-H and the -OH groups in BA-H, resulting in the grafting of vinyl groups to the polyacrylate copolymer chains. Apart from the postgrafting reaction between IPDI-H and BA-H, the thermal-curing process and the constrained movement of chain segments led to the formation of network structures with a relatively low cross-linking density. At this stage, UDAA exhibited adhesion properties. FTIR, time-test oscillation, and DSC were utilized to verify the progress of the postgrafting reactions.

**Figure 7:**
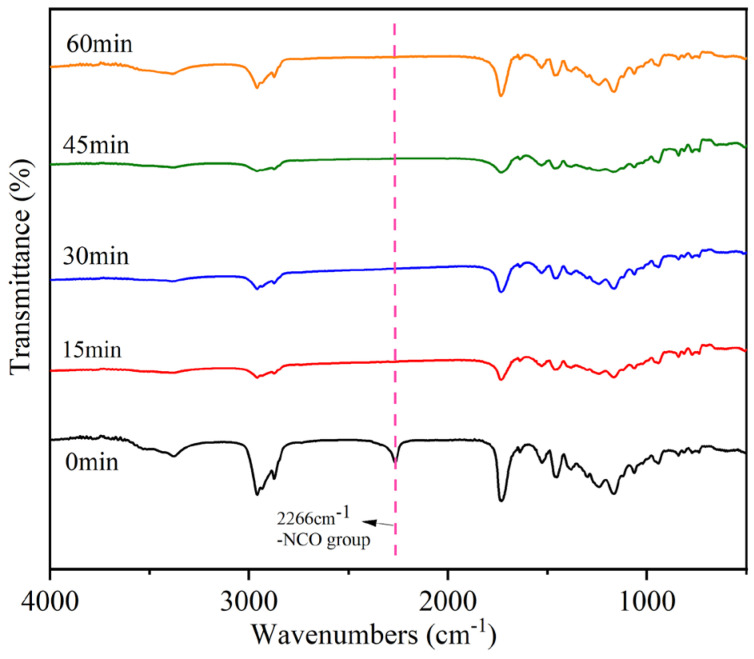
Infrared monitoring of the postgrafting reactions.

The postgrafting reactions were closely monitored using IR spectroscopy, and the corresponding results are illustrated in Figure 7. Samples were taken at 15-min intervals to enable comprehensive characterization. To ensure that the reactions were fully completed, a monitoring duration of 1 h was selected. In the IR spectra, a distinct peak at 2266 cm^−1^ was observed, which is attributed to the -N=C=O group [36]. This peak was clearly visible when the reaction had not yet commenced. However, it disappeared in the IR spectrum of the sample taken at 15 min, indicating complete consumption and reaction of the -NCO group. The subsequent sample’s IR diagram also exhibited the absence of the characteristic absorption peak associated with the -NCO group. Conversely, the presence of a peak at 3375 cm^−1^ indicates the existence of unreacted -OH groups within the system, which can potentially undergo further reaction with the active monomer. Based on the findings obtained from FTIR analysis, it can be concluded that the postgrafting reaction successfully achieved its intended objectives, namely, the consumption of the isocyanate group and a short reaction time.

**Figure 8:**
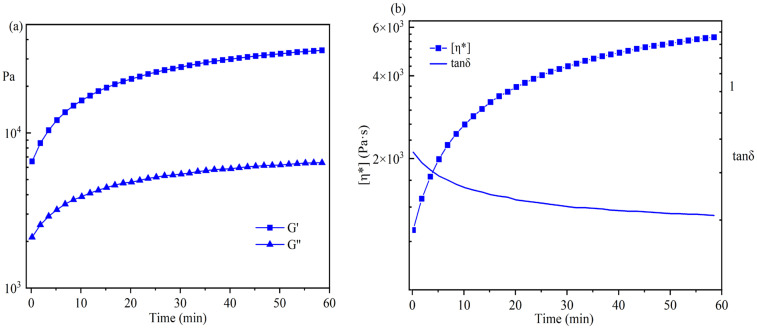
Time test oscillation monitoring results for the postgrafting reactions.

In Figure 8a, the changes in G′ and G″ were monitored by the time test oscillation during the postgrafting reaction. Figure 6a demonstrates that both the storage modulus (G′) and the loss modulus (G″) gradually increased during the postgrafting reaction, with G′ being greater than G″. There was no intersection point observed between them, suggesting that the sample’s structure remained unchanged. Initially, the system contained a higher concentration of small molecular weight IPDI-H before the postgrafting reaction commenced, leading to low values of G′ and G″. As the reaction progressed, the vinyl groups of IPDI-H were grafted onto the polyacrylate copolymer chains, resulting in an increased number of long-chain structures and a reduction in small molecular substances. Additionally, the branching chains within the system increased, resulting in easier entanglement. These entanglements contributed to an increase in the rigidity of the molecular chains [37]. Upon the completion of the reaction, the molecular weight of the system increased, making it more inclined towards the properties of viscoelastic solids.

In Figure 8b, the changes in η* and tanδ during the postgrafting reaction are depicted. The complex viscosity (η*) gradually increased as the reaction proceeded. This increase was attributed to both the decrease in solvent volume within the reaction and the presence of long-chain structures within the system, which enhanced the likelihood of chain entanglement and reduced the intermolecular distance. As a result, the intermolecular forces improved, leading to an increase in viscosity and a decrease in fluidity. Furthermore, the tanδ value decreased, indicating an increase in material elasticity and the gradual acquisition of elastomer properties. These findings aligned with the observed changes in G′ and G″.

**Figure 9:**
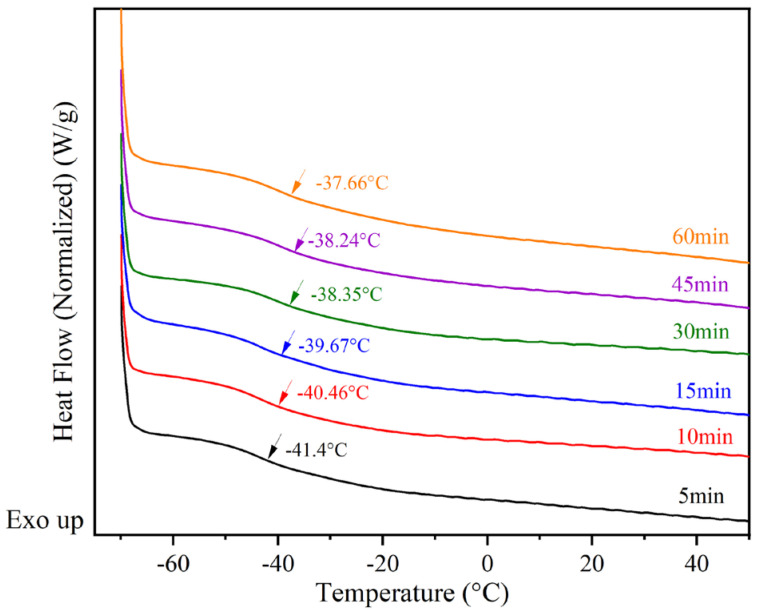
Differential scanning calorimetry (DSC) monitors Tg changes in the postgrafting reactions.

In Figure 9, the glass transition temperatures of the samples were monitored during the postgrafting reaction using DSC. As depicted in Figure 9, it can be observed that Tg gradually increases as the reaction proceeds. This can be attributed to two factors. Firstly, the vinyl groups attached to the main chains of BA-H contribute to the increase in glass transition temperatures, possibly due to a volume effect. Secondly, the samples undergo crosslinking and form network structures after the postgrafting reaction and thermal curing. This leads to a reduction in free volume and restricts the movement of the chain segments, resulting in a decrease in flexibility and an increase in Tg [38]. Based on these test results, it was determined that the postgrafting reaction can be completed within a few minutes. To ensure thorough reaction completion, a reaction time of 30 min was selected for this research.

### 3.3. Preparation of UDAAs

**Figure 10:**
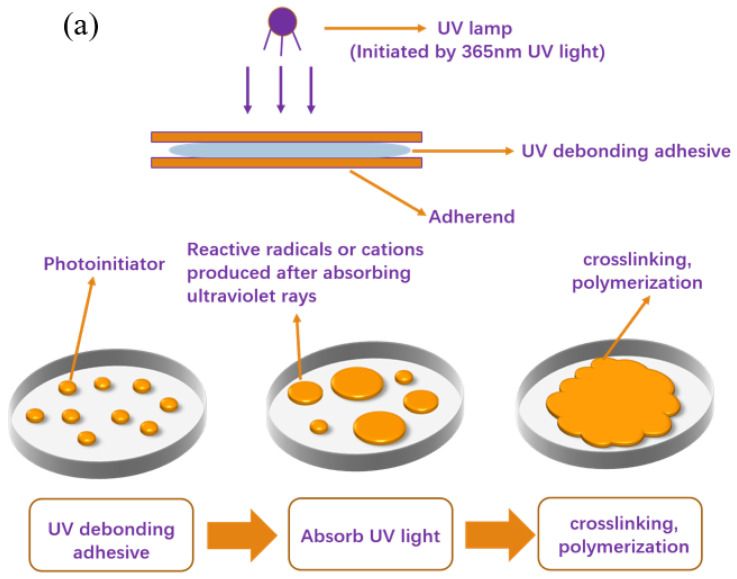

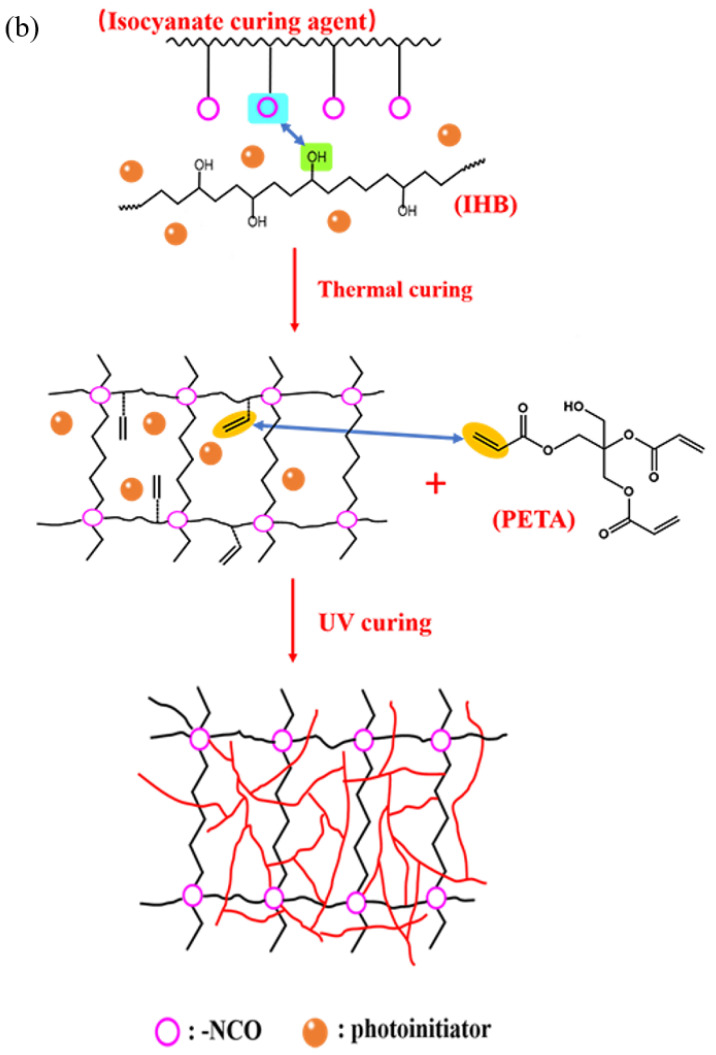
Schematic diagrams of (**a**) experimental operation and (**b**) debonding mechanisms of UDAAs during the UV radiation process.

Figure 10 illustrates the schematic procedure for the photoinduced formation of polymer networks in the acrylate debonding adhesive, specifically depicting the debonding mechanism of UDAAs. As depicted in Figure 10a, during the UV curing process, the acrylate UV debonding adhesives are irradiated with 365 nm ultraviolet light (ST2KW400MM, 100 mW/cm^2^), causing the photoinitiator to generate reactive radicals. These radicals initiate reactions between the double bond structures in the acrylate UV debonding adhesives and the double bonds in PETA, resulting in the formation of network structures characterized by a high cross-link density. This cross-linking effect induces significant volume shrinkage, which in turn leads to the development of wrinkles and micropores at the interface between the adhesive and the adherend. Consequently, the bonding interface is compromised, resulting in a reduction in bonding strength [39,40]. Furthermore, an increased crosslinking density causes the adhesive layer to harden, rendering the polymer network inflexible and impairing its adhesion properties. Figure 10b presents a schematic diagram illustrating the reaction of the acrylate UV debonding adhesive. In this step, the unreacted -OH groups in the primary acrylate copolymer chains are crosslinked with an isocyanate curing agent. Importantly, this process necessitates a low cross-linking density to preserve the PSAs’ adhesion properties. Subsequently, the reactive monomer PETA is introduced into the system. The double bond structures of the acrylate UV debonding adhesive interact with the double bonds in PETA, leading to the formation of network structures with a high crosslink density. As a result, volume shrinkage, wrinkles, and micropores manifest at the bonding interface, and the adhesive layer undergoes significant hardening, consequently resulting in a loss of viscosity.

**Figure 11:**
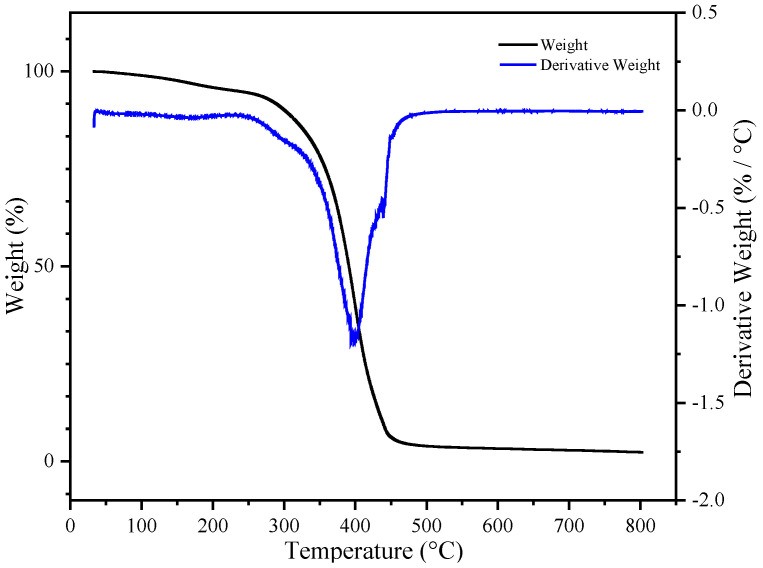
The thermogravimetry analysis (TGA) curve of UDAA.

Figure 11 shows the thermogravimetric curve of the final product, UDAA. Compared to the aforementioned BA-H, the final product, UDAA, contains a large amount of other substances with lower molecular weights. For example, tackifiers, special monomers such as PETA, IPDI-H, etc. The addition of these substances led to a decrease in the thermal stability of the product. Despite the addition of these substances, which resulted in decreased thermal stability, the weight loss of the final product was only 2.4% at 150 °C. This indicates the thermal stability of this product can still meet basic usage needs.

**Figure 12:**
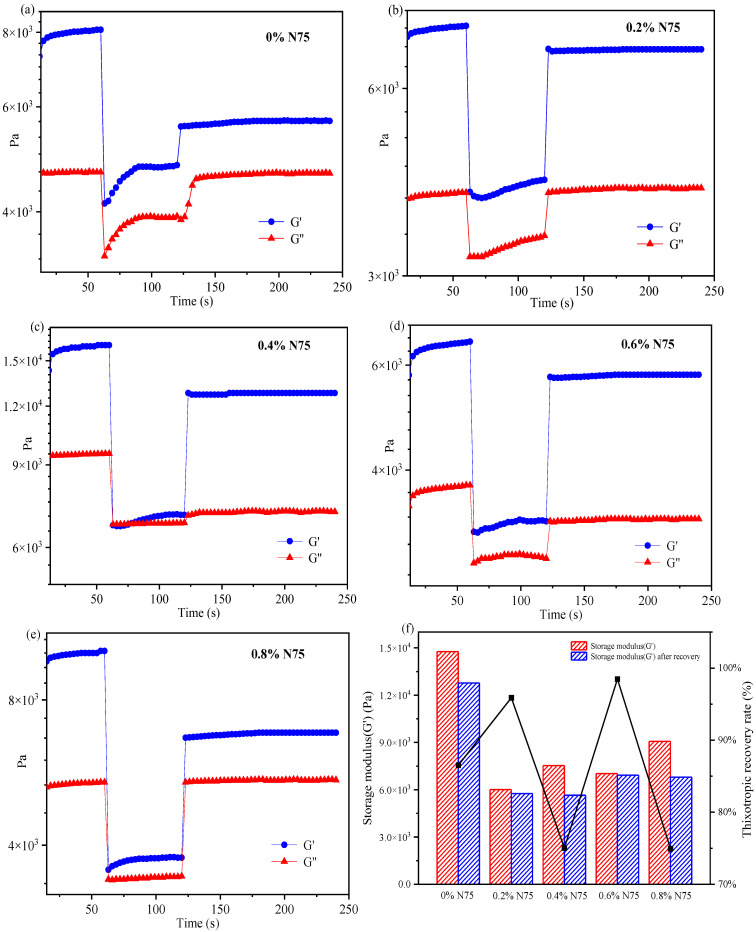
The thixotropic recovery performance of acrylate UV debonding adhesives with different contents of isocyanate curing agent (Desmodur N75).

To investigate the deformation and recovery behavior of acrylate UV debonding adhesive with varying concentrations of heat curing agent, the 3ITT mode in the rheometer was employed. Figure 12 illustrates the thixotropic recovery performance of acrylate adhesives with and without the isocyanate curing agent. The specific compositions for creating samples with different amounts of isocyanate curing agent can be found in Table 1.

In Figure 12, all curves indicate that G′ > G″, suggesting that the elastic properties of acrylate adhesives dominate over the viscous properties. In interval 2, a sharp decrease in the G’ value is observed. The sample, containing 0.4 wt% N75 (Figure 12c), breaks under high shear stress and cannot fully recover to its original state. By comparing intervals 1 and 3, the recovery rate is approximately 76%. When the addition of the isocyanate curing agent is 0.8 wt% (Figure 12e), the thixotropic recovery rate is similar to that of 0.4 wt%. Samples containing 0 wt% N75 (Figure 12a) and 0.8 wt% N75 have a low thixotropic recovery rate, which is susceptible to being damaged by external high shear stress and has poor thixotropic recovery performance. The adhesive with 0.6 wt% N75 (Figure 12d) is resistant to damage from external high shear stress and has a shorter recovery time, with the thixotropic recovery approaching 100%. Figure 12f provides a detailed record of the thixotropic recovery rate for each sample. As can be seen from Figure 12f, the thixotropic recovery rate of the sample adding 0.6 wt% N75 is the best, which is close to 100%. In summary, the deformation and recovery of acrylate UV debonding adhesives in the 3ITT mode offer a simple and convenient method to evaluate the adhesive’s performance. This experiment reveals that the deformation and recovery performance of acrylate UV debonding adhesives can be achieved by adding 0.6 wt% of the isocyanate curing agent.

**Figure 13:**
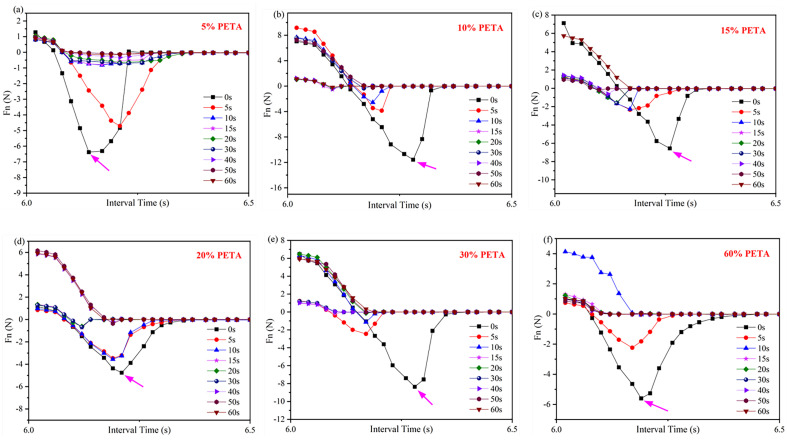
Tack test analysis of acrylate UV debonding adhesive adding (**a**) 5, (**b**) 10, (**c**) 15, (**d**) 20, (**e**) 30, and (**f**) 60 wt% reactive monomer (PETA), samples were irradiated for 0, 5, 10, 15, 20, 30, 40, 50, and 60 s at 100 mW/cm^2^. The positive and negative values of the Fn value only indicates the direction of Fn, with upwards as the positive direction.

Table 2 presents the maximum Fn values of acrylate UV debonding adhesives obtained by adding different amounts of the reactive monomer PETA under ultraviolet irradiation at various times. Figure 13 illustrates the tack test of acrylate UV debonding adhesives with varying contents of the monomer PETA. The negative force values in Figure 13 only represent the direction of Fn, and the time interval on the horizontal axis is 0.02 s. Additionally, Table 2 summarizes the data from Figure 13, providing a more intuitive representation of the data in Figure 13.

The tack test of samples under ultraviolet irradiation at different times reflects the adhesion and debonding properties of acrylate UV debonding adhesives. In Figure 13, the Fn values show a decreasing trend with increasing UV irradiation time. For example, when PETA is added at a concentration of 5 wt%, the maximum Fn value is 6.38 N before UV light irradiation. After 50 s of irradiation, the Fn value rapidly drops to 0.13 N, resulting in a sharp decrease in bonding strength and making it easy to peel off. This is because the photoinitiator (or photosensitizer) in the acrylate UV debonding adhesive, upon absorbing ultraviolet light, generates active free radicals or cations, leading to monomer polymerization and cross-linking. This conversion from a liquid to a solid-state occurs within seconds, causing the surface to harden and become inactive.

For acrylate UV debonding adhesive, the Fn value tends to initially increase and then decrease with the addition of PETA. This is because PETA contains unsaturated double bonds, which enhance the crosslinking and cohesion of the system, thereby improving peel strength. However, an excessive amount of PETA results in excessive double bonds and high curing shrinkage during the double bond curing process. The internal stress generated by volume shrinkage cannot be released in time, leading to a decrease in peel strength.

Figure 13 indicates that the maximum Fn value of adhesives before UV irradiation is 11.57 N. When the PETA content exceeds 30 wt%, the sample exhibits clean debonding behavior. After 50 s of irradiation, the Fn value approaches 0 N. At this point, adding an excessive amount of PETA will not benefit the strength of acrylate UV debonding adhesives. By combining 3ITT and tack test modes, the adhesive with 0.6 wt% isocyanate curing agent and 10 wt% PETA monomer is considered the optimal formula, according to the rheometer.

**Figure 14:**
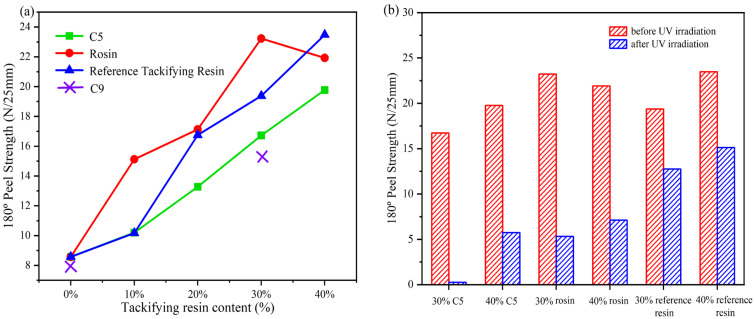
(**a**) peel strength of samples with different types of tackifying resins at different contents; ((**b**) Peel strength before and after UV irradiation with different types and contents of tackifier resins).

Table 3 presents formulations for the preparation of acrylate UV debonding adhesives with varying contents of a single tackifying resin. Figure 14 illustrates the peel strength of adhesives with different types of tackifying resins and varying contents.

Figure 14a illustrates that the peel strength of acrylate UV debonding adhesives increases with the addition of tackifying resin. However, when C9 was mixed with the adhesive during the experiment, it turned white, indicating that C9 is not compatible with the adhesive. When the content of the reference tackifying resin and C5 exceeded 30 wt%, the peel strength of the samples continued to increase. However, the effectiveness of the samples with the reference tackifying resin decreased after UV irradiation. Additionally, samples with rosin tackifying resin showed a significant decrease in bond strength at additions greater than 30 wt%. This is because when rosin is mixed with the acrylate copolymer, it improves the wetting performance and adhesive force, leading to increased peel strength. However, if the amount of rosin added is too large, the cohesive energy of the adhesive decreases, and the adhesive layer gets destroyed [41]. On the other hand, Figure 14b shows that the peel strength increases with the content of tackifying resin, and the peel strength of the acrylate UV debonding adhesive improves after UV exposure. However, this improvement is detrimental to the debonding performance of the adhesive in subsequent experiments.

**Figure 15:**
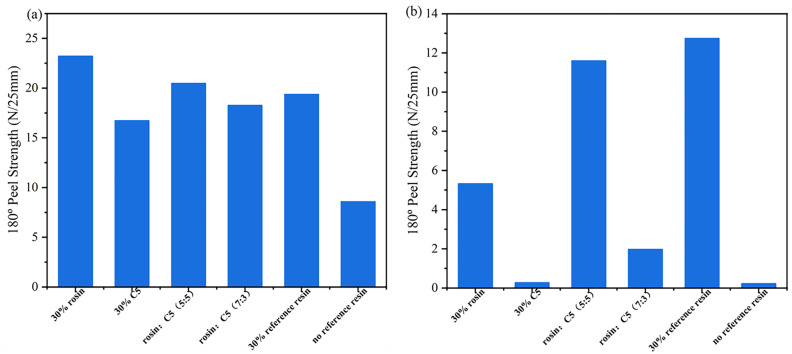
Peel strength of the samples containing compound tackifying resins: (**a**) before UV irradiation; (**b**) after UV irradiation.

Table 4 presents formulations for the preparation of acrylate UV debonding adhesives containing compound tackifying resins. Figure 15 is a graph depicting the results of the peel strength test of acrylate UV debonding adhesives with different tackifying resin ratios before and after ultraviolet irradiation.

As shown in Figure 15a, the adhesion properties were significantly improved by adding rosin resin individually. When using a combination of tackifying resins, the highest increase in adhesive adhesion was observed when the mixing ratio of 30 wt% rosin resin and 30 wt% C5 resin was 5:5. Figure 15b presents the analysis of the peel strength for acrylate UV debonding adhesives with varying proportions of tackifying resins after UV irradiation. It can be observed that the single reference tackifying resin had the poorest debonding performance, with rosin resin being less effective than C5 resin and C5 resin being slightly inferior to the pure tackifying resin. However, for samples containing compound tackifying resins, a better debonding effect was achieved when 30 wt% rosin and 30 wt% C5 resin were mixed at a ratio of 7:3, compared to a ratio of 5:5. In conclusion, the adhesive with 30 wt% C5 tackifying resin is considered the optimal formula.

**Figure 16:**
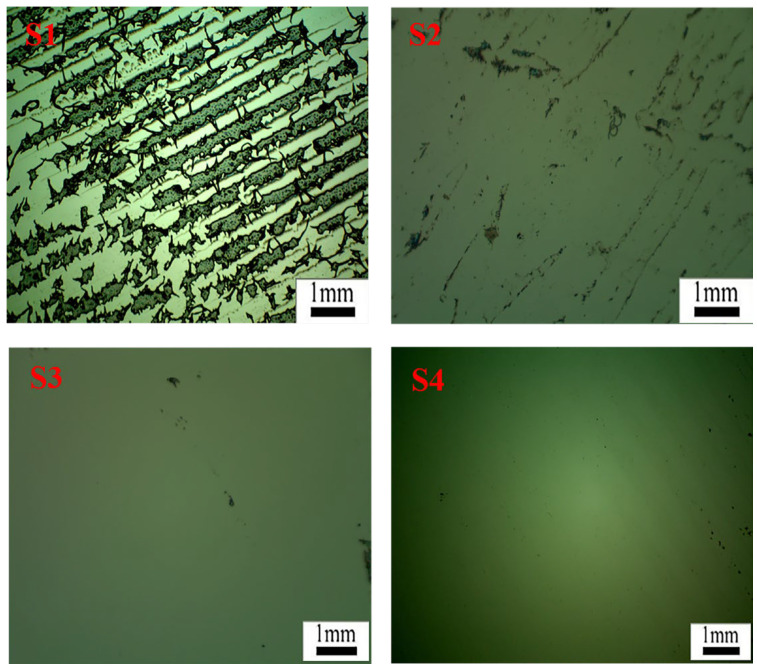
In light micrographs of the residue on the surface of the wafer after peeling the PET film with UV light at 100 mW/cm^2^ for 0, 5, 40, and 60 s (shown in **S1**–**S4**, respectively), the magnification is 50×.

Figure 16 displays microscope images of the silicon wafer surface after the removal of PET film coated with acrylate UV debonding adhesive under different UV irradiation durations. The black regions represent the residual adhesive remaining on the wafer surface. In S1, without UV curing, numerous residual regions can be observed, indicating that a significant amount of acrylate UV debonding adhesive remains without UV curing when peeling off the PET adhesive tape from the wafer surface. The microscope image in S2, after 5 s of UV curing, shows a substantial reduction in the adhesives remaining on the silicon wafer compared to S1. This reduction is attributed to the shrinkage of the adhesive volume during UV curing, resulting in a reduced bonding area. For 40 s or 60 s of UV curing, the black regions on the wafer surface in S3 or S4 nearly disappear, indicating that under 365 nm UV irradiation, the adhesives exhibit excellent peeling performance due to the design of vinyl groups attached to the main chains of acrylate copolymers through postgrafting reactions [2,42].

## 4. Conclusions

In this study, the vinyl groups of IPDI-H were successfully attached to the polyacrylate copolymer chains via postgrafting reactions. These reactions were characterized using FTIR, rheological methods, and DSC. The storage modulus, loss modulus, Fn values, and thixotropic recovery properties of UDAAs were tested before and after UV irradiation. It was discovered that a 0.6 wt% isocyanate curing agent improved the adhesive’s resistance to deformation, as demonstrated by the 3ITT test. Additionally, the tack test results indicated that a 10 wt% PETA monomer could withstand both adhesion and debonding properties. Moreover, the peel strength test suggested that the adhesion performance of acrylate adhesives increased with higher contents of tackifying resin. The optimal formulation was achieved by adding 30 wt% C5 tackifying resin. Microscope images of the wafer surfaces after peeling off the adhesive tapes revealed clean UV debonding properties after 40 s of UV irradiation, achieved by ensuring that the vinyl groups were attached along the BA-H main chains. In this research, acrylate UV debonding adhesives were prepared by grafting vinyls to copolymer main chains. UDAA has a high bonding force (≥15 N/25 mm) before ultraviolet irradiation, which can ensure the stability of products during production and processing. After UV irradiation, the bonding strength of UDAA decreased sharply (≤0.5 N/25 mm), and it can be easily peeled off so that it would not cause damage to the products and there would be no residual adhesives on the top of the products. This work significantly contributes to the development of UDAAs.

## Figures and Tables

**Table 1:** Formulations for the preparation of samples with different contents of isocyanate curing agent N75.

Sample	BA-H/g	IPDI-H/g	Isocyanate Curing Agent/wt%	PETA/wt%	Photoinitator/wt%
0% N75	20	2.08	0	10	2
0.2% N75	20	2.08	0.2	10	2
0.4% N75	20	2.08	0.4	10	2
0.6% N75	20	2.08	0.6	10	2
0.8% N75	20	2.08	0.8	10	2

**Table 2:** Maximum adhesion values of acrylate UV debonding adhesives by adding different contents of PETA after UV irradiation at different times.

	PETA	5 wt%	10 wt%	15 wt%	20 wt%	30 wt%	60 wt%
Fn/N							
0 s		6.38	11.57	6.56	4.76	8.36	5.6
5 s		4.71	3.86	2.34	3.46	2.45	2.24
10 s		0.82	2.56	2.27	3.56	1.13	0.05
15 s		0.79	0.53	1.65	0.72	0.19	0.03
20 s		0.65	0.34	1.57	0.61	0.14	0.02
30 s		0.71	0.36	1.63	0.68	0.05	0.03
40 s		0.32	0.41	0.62	0.21	0.03	0.04
50 s		0.13	0.23	0.18	0.36	0.04	0.02
60 s		0.17	0.19	0.06	0.03	0.02	0.03

**Table 3:** Formulations for the preparation of samples with different amounts of a single tackifying resin.

Samples	Types of Tackifying Resins	Tackifying Resin Content (wt%)
1	Reference tackifying resin	0
2		10
3		20
4		30
5		40
6	Rosin tackifying resin	10
7		20
8		30
9		40
10	C5 tackifying resin	10
11		20
12		30
13	C9 tackifying resin	30

**Table 4:** Recipes for the preparation of acrylate UV debonding adhesives containing compound tackifying resins.

Samples	30 wt% Rosin Tackifying Resin (Number of Parts)	30 wt% C5 Tackifying Resin (Number of Parts)	30 wt% Reference Tackifying Resin (Number of Parts)
1	10	0	0
2	0	10	0
3	5	5	0
4	7	3	0
5	0	0	10
6	0	0	0

## Data Availability

Data available in this paper after publication.
